# Conducting Health Literacy Research With Hard-to-Reach Regional Culturally and Linguistically Diverse Populations: Evaluation Study of Recruitment and Retention Methods Before and During COVID-19

**DOI:** 10.2196/26136

**Published:** 2021-11-02

**Authors:** Genevieve Perrins, Tabassum Ferdous, Dawn Hay, Bobby Harreveld, Kerry Reid-Searl

**Affiliations:** 1 Central Queensland Multicultural Association CQUniversity Rockhampton North Rockhampton Australia; 2 Centre for Research in Equity, Advancement of Teaching & Education School of Education and the Arts Central Queensland University Rockhampton Australia; 3 School of Nursing, Midwifery & Social Science Central Queensland University Rockhampton Australia

**Keywords:** health literacy, cultural and linguistic diversity, COVID-19, health care barriers, hard-to-reach research participants, regional Australia, health literacy profiles, literacy

## Abstract

**Background:**

In health research, culturally and linguistically diverse (CALD) health care consumers are cited as hidden or hard to reach. This paper evaluates the approach used by researchers to attract and retain hard-to-reach CALD research participants for a study investigating health communication barriers between CALD health care users and health care professionals in regional Australia. As the study was taking place during the COVID-19 pandemic, subsequent restrictions emerged. Thus, recruitment and retention methods were adapted. This evaluation considered the effectiveness of recruitment and retention used throughout the pre-COVID and during-COVID periods.

**Objective:**

This evaluation sought to determine the effectiveness of recruitment and retention efforts of researchers during a study that targeted regional hard-to-reach CALD participants.

**Methods:**

Recruitment and retention methods were categorized into the following 5 phases: recruitment, preintervention data collection, intervention, postintervention data collection, and interviews. To compare the methods used by researchers, recruitment and retention rates were divided into pre-COVID and during-COVID periods. Thereafter, in-depth reflections of the methods employed within this study were made.

**Results:**

This paper provides results relating to participant recruitment and retainment over the course of 5 research phases that occurred before and during COVID. During the pre-COVID recruitment phase, 22 participants were recruited. Of these participants, 15 (68%) transitioned to the next phase and completed the initial data collection phase. By contrast, 18 participants completed the during-COVID recruitment phase, with 13 (72%) continuing to the next phase. The success rate of the intervention phase in the pre-COVID period was 93% (14/15), compared with 84.6% (11/13) in the during-COVID period. Lastly, 93% (13/14) of participants completed the postintervention data collection in the pre-COVID period, compared with 91% (10/11) in the during-COVID period. In total, 40 participants took part in the initial data collection phase, with 23 (58%) completing the 5 research phases. Owing to the small sample size, it was not determined if there was any statistical significance between the groups (pre- and during-COVID periods).

**Conclusions:**

The success of this program in recruiting and maintaining regional hard-to-reach CALD populations was preserved over the pre- and during-COVID periods. The pandemic required researchers to adjust study methods, thereby inadvertently contributing to the recruitment and retention success of the project. The maintenance of participants during this period was due to flexibility offered by researchers through adaptive methods, such as the use of cultural gatekeepers, increased visibility of CALD researchers, and use of digital platforms. The major findings of this evaluation are 2-fold. First, increased diversity in the research sample required a high level of flexibility from researchers, meaning that such projects may be more resource intensive. Second, community organizations presented a valuable opportunity to connect with potential hard-to-reach research participants.

## Introduction

### Background

Low consumer health literacy levels represent an ongoing challenge to health services. Lower health literacy impacts an individual’s ability to access appropriate health services, comprehend medical instruction, and manage their own health [1]. In this way, individuals with low health literacy may forego preventative and proactive management of their health, leading to increased hospitalizations and presentation at emergency departments [1]. Despite the systemic support for promoting active and engaged health care consumers [2], the reality is that certain population subgroups have more difficulty in communicating their needs, engaging with health care professionals, and navigating the health care system. Culturally and linguistically diverse (CALD) health care consumers are at greater risk of lower health literacy levels due their social, cultural, geographic, and economic contexts [3].

Despite the importance of researching the experiences of CALD health care users, this demographic is often cited as hidden or hard to reach [4]. The reasons for this categorization are multiple; migrants may be socially disconnected, may be vulnerable, may have a fear of discrimination, may misinterpret or misunderstand the research process, or may generally distrust researchers [4]. Therefore, there are significant difficulties in recruiting and retaining such a research cohort. In the pursuit of meaningful research in this area, this paper provides valuable insights into the recruitment and retainment of traditionally hard-to-reach research populations [4].

During the research project, COVID-19 emerged. As a result, recruitment and retention methods had to be adapted to address corresponding lockdowns and restrictions. Researchers analyzed the recruitment and retention methods by breaking down the participation process into 5 distinct phases and comparing pre-COVID and during-COVID approaches for each phase. These phases include recruitment, preintervention data collection, intervention, postintervention data collection, and interviews.

### Objectives

The objective of this study was to provide insights into recruitment and retention methods used during a study targeting a highly diverse hard-to-reach CALD sample in a regional city in Australia.

## Methods

### Study Design

The research project was conducted by a community organization based in the central Queensland city of Rockhampton. The organization, Central Queensland Multicultural Association, offers an eclectic range of community programs aimed at overcoming social isolation and promoting integration and engagement. This pilot project was funded by the Australian Government through the Department of Health, for the purposes of (1) establishing an evidence-based health literacy profile for CALD populations residing within the Rockhampton area and (2) examining the efficacy of community-based education sessions as a health intervention option to address ongoing health literacy issues identified from health literacy profiling outcomes.

### Participation

While the study sought input from both health care consumers and professionals to establish a health literacy profile, this paper specifically reflects on the difficulties and successes of recruiting CALD health care consumers (herein referred to as participants).

**Figure 1:**
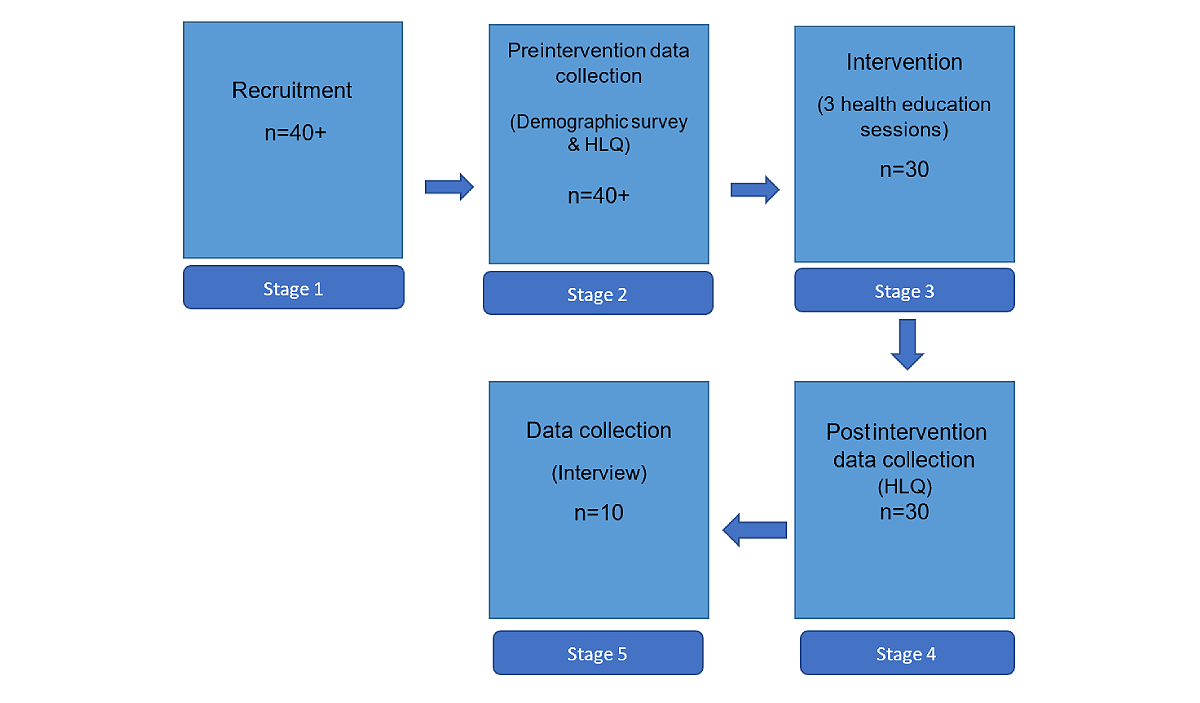
Target number of research participants at different phases of the project. HLQ: Health Literacy Questionnaire.

As illustrated in Figure 1, the process of consumer participation included a recruitment phase, preintervention data collection, an intervention phase, and postintervention data collection. Thereafter, 10 participants were invited to give interviews to provide further qualitative data.

So as not to place heavy restrictions on recruitment, consumer participant criteria were left sufficiently broad. Participants were required to be over the age of 18 years, have moved to Australia from another country, be Medicare eligible, and have adequate English ability.

### Quantitative Research Tools

The research program utilized the prevalidated Health Literacy Questionnaire (HLQ) designed by Osborne et al [5]. The HLQ aims to test consumers’ self-reported capability for conducting health literacy tasks. The HLQ includes 21 questions that feature Likert-scale responses.

### Qualitative Research Tools

Researchers designed an interview schedule that reflected the domains of health literacy set out within the quantitative portion of the study. The schedule included 11 questions relating to the HLQ domains, which were designed as a start point for conducting semistructured interviews.

### Intervention

The program’s intervention involved 3 education sessions delivered by a researcher. The intervention was designed to (1) inform CALD health consumers about their role in interactions with health care providers, (2) increase consumer understanding and manage expectations of clinical appointments, and (3) inform participants about local health system navigation. Within the education sessions, additional resources were utilized to aid participant understanding, including PowerPoint, as well as videos and resource sheets published by government agencies.

### Evaluation Tools

Participants were observed in their dealings with researchers. Researchers made note of participant behavior, including avoidance, requests to withdraw, and connection-seeking, as well as preferences associated with location and group makeup. These interactions provided important insights into participants’ feelings toward the research process, including (1) the perceived significance of the research to the individual and the community, (2) whether it was considered a justified use of time, (3) the need for having social support during the research process, and (4) distrust or anxiety.

These observations were recorded in a research journal and reflected on in meetings between researchers, as well as in discussions with community leaders. The use of observations was a necessary data collection method as researchers had to adjust recruitment and retention methods in response to these observations.

In addition to observations, the program sought participant feedback in order to evaluate the effectiveness of the program’s recruitment and retention methods. Data collection methods included the use of an online survey or interview questions. The use of either of these data collection methods was determined by the participants’ levels of involvement in the project. For example, if a participant had agreed to be interviewed as part of the research project, researchers would include evaluative questions as part of the interview. However, if participants had not taken part in an interview, had initially signed up but later withdrawn, or failed to complete all the steps for participation, they were sent an online survey. Both data collection methods queried the same potential issues associated with recruitment or retention. These included (1) logistical issues such as transport and childcare, (2) time constraints, (3) the impact of session size and group makeup, and (4) the participation processes.

### Evaluation Analysis

Researchers analyzed the responses provided by participants in their interviews and online surveys in order to gather information on what barriers were faced by participants during the research process.

In addition to direct feedback and observation, researchers analyzed the dropout rates of participants within the 5 research phases. The recruitment phase refers to “the dialogue which takes place between an investigator and a potential participant prior to the initiation of the consent process” [6]. The process includes the identification of potential participants, filtration, and discussion of participation steps. The preintervention data collection phase refers to participants completing necessary paperwork to take part in the study. This paperwork included a program consent form, a demographic survey, and a preintervention HLQ. The intervention stage refers to the 3 education sessions (S1, S2, and S3). The postintervention data collection phase refers to the collection of completed HLQs following the 3 education sessions. The interview phase refers to the completion of a one-on-one semistructured interview with researchers.

As each of these participation phases occurred within pre-COVID and during-COVID periods, researchers were able to adapt recruitment and retention methods, therefore providing a basis for a comparative analysis. Researchers then compared the dropout rates for each participation phase within each designated period (pre-COVID or during-COVID period).

### Ethics

Engagement with health care consumers was approved on an ethical basis by the Central Queensland Hospital and Health Service Human Research Ethics Committee (reference: LNR/2019/QCQ/57544).

## Results

Table 1 provides a breakdown of the research project’s recruitment and retention results according to the COVID period and research phase.

**Table 1:** Number of participants before and during COVID-19 restrictions at different phases of the study.

Period	Recruitment (N=40), n (rate)	Initial data collection: demographic and HLQ^a^ (N=28), n (%)	Intervention			Postintervention data collection: HLQ (N=23), n (%)		Interview (N=10), n	
			Sessions 1 and 2 (N=28), n	Session 3 (N=25), n (%)					
Before COVID-19 restriction (November 30, 2019, to January 30, 2020)	22 (2.9/week)	15 (68%)	15	14 (93%)	13 (93%)		0		
During COVID-19 restriction (January 31, 2020, to August 7, 2020)	18 (0.66/week)	13 (72%)	13	11 (85%)	10 (91%)		10		

^a^HLQ: Health Literacy Questionnaire.

## Discussion

### Recruitment

A major recruitment success was the diversity of the research sample. The 40 participants who took part had 15 separate countries of origin. Participant country of origin and world region are presented in Table 2.

**Table 2:** Participant profile by world region and country of origin.

World region	Country	Participants (N=40), n (%)
Southern Asia	Nepal	8 (20)
Southern Asia	Bangladesh	6 (15)
Southern Asia	Sri Lanka	5 (13)
Middle East	Afghanistan	4 (10)
Mainland South-East Asia	Myanmar	4 (10)
Melanesia	Papua New Guinea	4 (10)
South America	Brazil	1 (3)
Chinese Asia (includes Mongolia)	China	1 (3)
Northern Europe	Denmark	1 (3)
Polynesia (excludes Hawaii)	Fiji	1 (3)
Western Europe	Germany	1 (3)
Southern and East Africa	South Africa	1 (3)
Melanesia	Solomon Islands	1 (3)
Mainland South-East Asia	Thailand	1 (3)
Mainland South-East Asia	Vietnam	1 (3)

The greatest concentration of Queensland’s CALD health care users is in Brisbane; as of 2018, 30.6% of the capital’s population had a CALD background. However, there are increasing levels of diversity among the regions [7]. Rockhampton’s CALD population has been growing steadily; in 2011, 8.7% of the regional city’s population was CALD, compared with 9.4% in 2016 [7]. Further, as of 2016, 6.8% of Rockhampton’s population spoke a language other than English at home [8]. However, this is not to say that Rockhampton’s CALD population is necessarily homogenizing. Owing to governmental policy, which promotes migration to the region [9], the city’s migrant population is likely to become increasingly diverse. In contrast to studies of CALD health care consumers in metropolitan areas, research in regional areas must account for a significant amount of cultural diversity within the sample. Therefore, recruitment and retention methods that can be adapted to multiple cultural backgrounds may be more successful. In the case of this research program, the ability to recruit and retain such a diverse sample represents a success.

#### Pre-COVID Period

The program used purposive, convenience, and snowballing methods to recruit participants. Beyond participants who were recruited via broader means (eg, public community days discussed further under the Intervention subheading), researchers utilized immediate networks to gauge the interest of potential participants. The use of established community networks was of great benefit to researchers; it required a lesser degree of time and resources to locate potential participants, and researchers had already established a level of rapport through other dealings with community members.

While snowballing and convenience sampling methods in this project were largely effective in recruiting participants, overreliance on established networks can be problematic for the research objectives. Overreliance on such a network risks introducing self-selection bias, as willing participants may be well settled, educated, nonisolated, and interested in research [4]. In spite of this limitation, ethnic minorities are often difficult to recruit using traditional sampling methods owing to suspicion of research/researchers, vulnerability, social isolation, and stigma [4]. Although an acknowledged bias, other options for reaching hidden populations is yet to be discovered, and as such, limitations need to be endured in order to pursue much needed research in this area. Within this study, care was taken to ensure that snowballing starting points were as diversified as possible. As illustrated in Table 2, initial recruitment and potential networks were selected by country of origin to ensure that broad geographic regions were accounted for.

To further diversify participant background, researchers also attempted to broaden the recruitment method by engaging with other community institutions and their leaders. Such organizations included language groups, and cultural and social organizations, as well as places of worship. There was significant potential in connecting with such organizations, and if successful, there was access to a high number of diverse participants. However, researchers found this approach to be ineffectual. While organizations had expressed an interest in connecting contacts with researchers, in each case, these attempts were unsuccessful. This approach was therefore found to be highly bureaucratic and resource intensive.

#### During-COVID Period

With COVID restrictions ceasing many day-to-day activities that researchers had previously used as a gateway to recruitment, as well as the difficulty in recruiting participants via institutional leaders, researchers sought out community leaders instead as the instigation point for recruitment. In this paper, institutional leaders refer to those who are paid, who hold positions of authority designated to them by way of a constitution/contract, or who hold sway over large groups of people not individually known to them. It is a formal position. Community leaders, by contrast, are informal positions not held within an institution, but they instead are figures who hold authority by way of their connection with individuals residing in the community. Often, community leaders are associated with specific religious, ethnic, or cultural groups.

It was recognized that a direct approach to individuals within the CALD community was not effective. It resulted in participant reluctance due to fear of the research process and researchers, as well as stigma. Instead, researchers decided to approach cultural and religious groups. In doing so, researchers would reach out to community leaders to gauge interest in the project. The project used the established network of the organization to identify and recruit community leaders. For example, the diversity of the organization’s management committee and workforce allowed researchers to tap into hidden South American populations, recruit socially isolated participants from within the local Mosque, and access a women’s group based around cultural diversity and inclusion activities. Further, the organization itself represented a drawcard to ethnic minorities, as these groups would seek out assistance relating to the organization’s broader function. Their attendance at the organization allowed researchers to approach for discussing the research program and participation.

Community leaders acted as research gatekeepers; they would explain the process of participation to potential participants, provide emotional support, and act as mediators between the researchers and participants. The use of gatekeepers in research is largely successful, particularly where the subject is deemed significant within the community [10]. The findings of this program were that the use of cultural/religious gatekeepers is highly effective.

In addition to the use of cultural gatekeepers, approaching participants within their cultural groups overcame recruitment issues. Such an approach worked well during COVID-19 restrictions, when opportunities for researchers to themselves establish contact and rapport were diminished. Researchers felt that community inclusion overcame issues of participant self-assuredness and made participation seem less daunting. Participants were more forthcoming in speaking of their experiences, they had a chance to build rapport with researchers in a less threatening environment, and it provided a level of anonymity for participants. From the researchers’ perspective, the increase in willingness from the side of the participants was attributable to both the use of intimate cultural groups and the use of cultural gatekeepers.

The shift from recruiting participants face to face at events to a purely network-based recruitment method meant that enlistment became more targeted. Difficulties in recruiting CALD participants were seemingly compounded when potential participants encountered an Anglo-Australian researcher. The researchers tasked with data collection were 2 women (one had an Anglo-Australian background, and other had a Bangladeshi background). Observational evidence acquired by the researchers suggested that the meeting of researcher and participant backgrounds was significant. In fact, in cases where age or gender disparities between an Anglo-Australian researcher and potential participants were minimal, the participants still gravitated toward researchers twice their age, but with similar religious or cultural backgrounds. The success rate of recruitment was variable, with more success when participants were faced with a researcher from a migrant background. This finding corresponds with previous literature, which identifies perceived power differentials as a major factor in the dearth of CALD individual participation in health research [11-13]. In response to these difficulties and the issues outlined in the research, adjustments to recruitment were made to ensure that the CALD researcher represented the “face” of the program, with participants enjoying maximum contact with this researcher where possible.

### Preintervention Data Collection

#### Pre-COVID Period

In order to minimize the dropout rate between recruitment and data collection, those who agreed to participate during the recruitment phase were presented immediately with data collection materials. This approach worked well when the research organization had agreements with cultural groups for the use of facilities. For example, the research organization would allow the use of rooms for language classes. Often when these classes were held, members of cultural groups would congregate. Researchers used the opportunity to recruit these participants and present them immediately with data collection materials. For establishing a health literacy profile for this region, this method was highly successful; 14 participants were initially approached, with 8 being eligible and successfully completing the preintervention data collection phase. Participants were happy to be approached and document their experience; however, this did not necessarily translate to high levels of participant retention. In the pre-COVID period, 8 participants were recruited in this fashion; however, only 3 of those were retained and eventually took part in the intervention phase. The dropout rate in this instance was hypothesized as participants being reluctant to return to complete the intervention. Researchers felt that for this research cohort, making the research process as seamless as possible may be more attractive than requiring multiple distinct steps to complete participation.

#### During-COVID Period

As normal activities conducted by the organization ceased under COVID restrictions, routine data collection processes were amended to ensure the feasibility of this research phase. The researchers’ recruitment pitch had to highlight the importance of the research objectives to not only the individuals, but also their broader community [14]. In the pre-COVID data collection phase, while this was still an important factor to be included, participants were easily swayed by the limited amount of time and resources necessary to participate. Throughout the during-COVID data collection phase, researchers were required to follow-up with participants to enquire about any difficulties, to arrange collection of the paperwork, and to discuss the next stages.

Even with the increased barriers to participation that COVID-19 posed, a continuance rate of 72% (13/18) during this period indicates that researchers successfully adapted their data collection approach so that it suited CALD research participants. This was done by offering a high level of flexibility, such as the collection of paperwork from residences or places of work (at the participant’s request), the use of digital platforms to deliver and receive completed questionnaires, and the ability of researchers to gather data collection tools at the upcoming intervention phase. Despite the level of flexibility, some participants were difficult to retain, and ceased contact with researchers. These participants did not provide feedback to researchers; however, it is believed that participant withdrawal in this period was due to potential time constraints and unawareness of the research process. Unfortunately, in cases where participants had ceased contact, the requirements of participation could not be explained.

### Intervention

#### Pre-COVID Period

The original approach was to hold sizeable community sessions, whereby a large number of participants could be provided with the first 2 sessions (S1 and S2) of the intervention. This approach not only satisfied program funding arrangements with respect to community engagement, but also allowed for minimal resource requirements in terms of staffing and time. It was initially anticipated that the program would require 3 to 4 of these large gatherings to collect consumer participant numbers. The first intervention took place on the community health day. The health day was advertised to the broader community, and no restrictions were placed on attendance. Researchers had arranged for local health professionals to attend and provide free health checks. Resource packs were provided to attendees with local health information, as well as navigational aids for the region’s health service. Further, the research organization arranged for childcare services for the duration of the health day. Researchers took the opportunity to identify potential participants who attended. Those who agreed to take part were presented with data collection materials before the intervention took place. Each of these sessions was held onsite, at the organization’s office.

Embedding the intervention within the community health day represented an effective way to engage potential participants. Many community members attended the intervention as part of the community health day but did not necessarily agree or were eligible to participate in the research project. The total number of attendees at community days was 23, with 10 being eligible and agreeing to participate. Of the 13 who did not participate, 7 were ineligible. Therefore, 6 were eligible but did not agree to participate. Researchers hypothesized that a lack of awareness of the research process and aims was to blame for the reluctance of eligible nonparticipants. However, because the intervention was delivered to sizable groups, it was not possible for researchers to identify potential problems and reassure reluctant nonparticipants. Of the 10 participants who agreed to participate during the community health days, 9 went on to complete the intervention in its entirety. Despite this reasonable success, subsequent attempts to organize the third portion of the intervention (S3) in this way were met with participant reluctance, and it was not possible to replicate the earlier success.

Researchers considered that this reluctance was due to education sessions that were presented too formally and with large culturally diverse groups. The formulation of education sessions was done with oversight from health science academics and staff from the health service. However, due to restraints associated with a pilot study (discussed further in the Limitations subsection), the education sessions were not formulated with community feedback. Subsequently, as the project responded to COVID restrictions and the delivery of sessions was to smaller culturally homogenous groups, feedback from participants suggested that informal delivery, including participant discussion and question time, was more impactful and more aligned with participant expectations.

The pre-COVID intervention was always delivered face-to-face. The choice to deliver the program directly to participants is symptomatic of a broader organizational approach to overcoming social isolation and enhancing integration. The success of the face-to-face approach was highly dependent on the individual. For example, some participants sought out the research program as a way to establish contact with the organization and community in order to overcome social isolation. These participants gained more from direct face-to-face contact with researchers than they would have from an online program. However, in other cases where participants were sufficiently integrated, had established themselves within the broader community, and had higher employment or familial commitments, face-to-face interaction represented a barrier to participation. Researchers were aware of the individual circumstances of each participant, and as such, would regularly hold sessions outside business hours, such as on weeknights or weekends, and they employed childcare services to ensure women could attend and were mentally present during the intervention. Both approaches were effective in overcoming barriers. Due to the effectiveness of these approaches, they were again utilized by the research team in the during-COVID intervention phase.

#### During-COVID Period

The emergence of COVID-19 brought with it restrictions on face-to-face events, which required alterations to session delivery within the program. Researchers initially minimized the number of participants present at each intervention session and shifted the venue from the research organization to one of participant choosing. The session size was 10 or over pre-COVID but had to be restricted to no more than five in each session. Researchers found that participant willingness to partake in the entire intervention process increased during this period. Participants indicated that smaller and more intimate sessions gave them the opportunity to speak about their experiences and allowed researchers to refine the intervention to suit particular individuals and their needs. While the numbers during this period may suggest a decrease in participation, subsequent discussions with participants suggested that sessions taking place under this method were more meaningful to each participant. Further, in holding sessions in spaces of participant choosing, the program effectively overcame potential barriers experienced by participants such as work commitments, transport, childcare, and time constraints.

During the height of restrictions, in which contact with persons outside the household was disallowed, the intervention continued via online platforms. Sessions 1 and 2 were delivered by a researcher via Zoom (Zoom Video Communications). The third session was delivered either by Zoom or via a prerecorded session uploaded to YouTube (Google Inc). This arrangement allowed for researchers to connect with participants, thereby increasing participant opportunity to speak of their experiences and ask questions while adhering to public health restrictions. In some cases, participants benefitted greatly from the flexibility. However, while the project maintained targeted numbers during this period, the relationship fostered online, compared with face-to-face, is more superficial. Research participants of diverse backgrounds have a greater need for social comfort in a research context [11]. Therefore, such needs risk not being met when researchers make participation a solely online activity.

### Postintervention Data Collection

#### Pre-COVID Period

Following successful completion of the intervention, participants were required to fill in a postintervention HLQ. Initially, researchers posted the HLQ to participants and requested them to return the paperwork either to the organization’s office, via post, or email. Researchers attempted to give sufficient consideration for individual circumstances; however, expecting some participants to return the paperwork increased barriers to participation. In particular, researchers noticed significant participant fatigue by this stage of the program, therefore requiring researchers to provide further flexibility to maintain participant numbers. To provide this flexibility, researchers collected the data collection materials from a location and at a time requested by the participants. The finding of the research team was that the initial approach in requiring participants to return their paperwork was largely ineffective. This is because it inadvertently placed further restrictions on participation, such as the need for transport and childcare, as well as needing to fit this responsibility between employment or familial obligations.

#### During-COVID Period

The collection of hard-copy postintervention data during COVID restrictions, particularly for participants who required assistance, was laborious. However, in cases where participants had been identified as sufficiently literate (both digitally and with forms) researchers used Google Forms as a way to disperse questionnaires. It was considered that reworking the paperwork into a Google Forms format was beneficial in that it may have appeared less daunting than the layout of the tangible form, while minimizing COVID-related and traditional barriers to participation. When virus restrictions allowed, the research team resumed collection of tangible forms.

The collection of tangible forms was preferable as it gave an opportunity to assist participants who faced difficulties in filling out the questionnaire. The finding of this project was that the presentation and language of the HLQ involved some difficulties. First, some participants suggested that they had not encountered a survey of this type (Likert scale) and mentioned that they were unsure about how to correctly fill in the form. Second, despite participants speaking English, some encountered difficulties with the language used in the data collection material.

### Interviews

After completing the quantitative aspect of the study, 10 participants were invited from the sample to take part in semistructured interviews. There were no further selection criteria to take part in the interview phase, instead researchers were opportunistic. The initial study design called for all quantitative data to be collected before interviews could take place. This was so that participants could build upon the answers provided in the survey, as well as supply the project with feedback about their experiences with the pilot program. For these reasons, the interview phase of the project only occurred throughout the during-COVID period, and therefore, success rates cannot be compared.

The researchers approached interviews on a flexible basis, often offering participants the choice of an online Zoom interview or an interview at a location of their choosing. Time frames were also flexible, with researchers making appointments according to participants’ availability (eg, weekends and nights). As the study only required 10 participants for the interviews, the slots were filled quickly and easily.

### Evaluation Limitations

Community-based interventions designed to address health literacy within population subgroups may undergo a process of needs assessment before intervention co-design and implementation [15]. Within this pilot study, it was not feasible to establish an intervention off the back of rigorous community engagement, instead this program sought input from the health service and academics within health sciences. This is a noted limitation; it is preferable to incorporate input from the target population in order to ensure intervention methods are appropriate and tailored [15]. If education sessions were formulated with more feedback from the intended population, participant recruitment and retention might have been enhanced.

Further, this pilot project did not employ translators or interpreters for participants. Researchers considered that the use of accredited interpreters and translators would diversify the sample by removing the need for participants to be proficient in English, as well as ensuring that interpretations were true to their original meaning. Considering the diversity of the research sample, the cost of translating materials and employing interpreters within this pilot study was prohibitive. To mitigate difficulties, researchers provided assistance and clarification when requested to do so. Researcher involvement with completion of the questionnaire has the potential to introduce bias; however, such a limitation had to be endured to be sure that certain participants understood what was asked and how to properly record their answers.

### Conclusions

This paper documented the efforts of researchers to recruit and retain CALD participants before and during COVID-19 restrictions. While it was anticipated by researchers that COVID would negatively impact the research program, alternative action in response to COVID actually assisted researchers in identifying the strengths and weaknesses of prior methods and gave an opportunity for researchers to evaluate the efficacy of different methods. The unforeseen pandemic inadvertently forced researchers to rethink the initial study design, which benefited the research aims.

There are a number of takeaways from this program. First, the sample used by this project was highly diverse. The sample included 40 individuals having 15 countries of origin. While other studies have targeted and discussed recruitment difficulties and successes as they relate to specific ethnic groups, there is a shortage of information relating to the recruitment of a research sample that involves a high level of cultural diversity. This study identified the way in which the term CALD obscures the true diversity of regional-based migrant populations and provided necessary insights into the way in which such a group can be incorporated into health research. Lastly, this project highlighted the way in which community-based organizations have a discernible role in connecting hard-to-reach participants with research projects.

In a study such as this, difficulties in recruitment and retention may have spurred similar actions irrespective of the occurrence of the pandemic. Therefore, in similar studies conducted outside the impact of the pandemic, recruitment and retention may look similar, that is, first attempts may aim to exhaust traditional methods, such as large-scale recruitment via community health days, large intervention sessions, and use of institutions to recruit community members, before turning toward tailored culture-specific bottom-up approaches. The initial success of the pre-COVID period, followed by recruitment stagnation, as well as the ongoing success of during-COVID procedures, suggests that a high level of diversity in the research sample requires a high level of diversity in recruitment and retention methods. Therefore, the keys to ensuring successful recruitment and retention are to provide flexibility; respond to individual and group circumstances; reflect on specific environmental, geographic, and socioeconomic barriers; and mitigate issues. Considering the heightened requirement for flexibility, a major takeaway from this study is that research focusing on CALD samples is inherently more time and resource intensive. Therefore, future studies that target such a population will need to factor this into the study design and cost.

## Abbreviations

CALD: culturally and linguistically diverse
HLQ: Health Literacy Questionnaire
